# The Book World of Medicine and Science

**Published:** 1896-02-15

**Authors:** 


### THE BOOK WORLD OF MEDICINE AND SCIENCE.

THE CHEMIST'S COMPENDIUM. By C. J. S. THOMPSON. (London: Whittaker and Co., 1896, pp. 230. 2s. 6d. net.)

We congratulate Mr. Thompson in condensing in a handy book of reference the formulæ of value to the practical pharmacist, and advise the purchase of this volume for daily use on the dispensing counter. No similar book exists of convenient size and in such compact form, and as the author has included the more modern remedies, it should prove of service to those who are often troubled with the posology of new drugs. We think that the section on milk analysis might have been omitted, as the chemists and druggists who will use this book are not called upon to do such work, and would undoubtedly come into conflict not only with Somerset House but also with public analysts if they attempted such analyses on the lines formulated by the author. The method suggested for estimating the total fats is long and cumbrous, and has long since been displaced by the Adams' coil process and other improved methods. We notice on p. 228 in the list of medicaments issued by the Board of Trade a few verbal inaccuracies, amongst them Juson's Liquid Disinfectant for Tuson's Liquid Disinfectant, and hope, although this is not the fault of the author, that the Board of Trade will soon see fit to revise this antiquated list of disinfectants and medicaments for the use of ships at sea. Urinalysis is an indifferent term used by the author for urine analysis, which might with advantage disappear from our dictionaries, and in his description of the sugar tests for urine he seems to have overlooked Allen and Johnstone's recent contributions to this branch of analysis.

GOUT AND RHEUMATIC GOUT. By Dr. ALFRED WRIGHT. (London: Simpkin, Marshall, and Co., 1895, pp. 43.)

This is what we may fairly call a watering-place book. It is admittedly addressed to patients rather than to the profession, and although not a bad little book in some respects, does not with any excessive reticence hide the moral which it teaches—to consult the author. Various measures are recommended, including seaweed, electricity, and a compound which the author calls "dumoline." A very comfortable diet is advised, and altogether we have no doubt that those who crave for "an 'up-to-date' treatment, free from irksome dietary and other restrictions," as it is described on the cover, will find in this pamphlet what they seek. Its success will probably depend much on idiosyncrasy.

OUR TREASURES, AND HOW TO KEEP THEM. (London: Liquor Carnis Co.)

The author of this little book of some hundred pages holds a brief for the Liquor Carnis Company, and while giving some useful hints on the dietetic management of infants, devotes most of his space to an exposition of the many virtues of virol as a substitute for, or supplement to, other forms of food for infants of three months and upwards. Although believing thoroughly in the excellence of this preparation, we should hesitate to recommend its universal adoption in uncomplicated and straightforward cases. We still believe that milk is the best food for the first year of infant life, although it may occasionally require certain simple modifications. The Liquor Carnis Company will forward a copy of this book to any of our readers on application to the secretary of the company, 28A, Farringdon Street, E.C.

